# The bacterial toxin CNF1 as a tool to induce retinal degeneration reminiscent of retinitis pigmentosa

**DOI:** 10.1038/srep35919

**Published:** 2016-10-24

**Authors:** Viviana Guadagni, Chiara Cerri, Ilaria Piano, Elena Novelli, Claudia Gargini, Carla Fiorentini, Matteo Caleo, Enrica Strettoi

**Affiliations:** 1 Neuroscience Institute, Italian National Research Council (CNR), Pisa, 56124, Italy; 2 Accademia dei Lincei, Rome, 00165, Italy; 3 Department of Pharmacy, University of Pisa, Pisa, 56126, Italy; 4 Istituto Superiore di Sanità, Rome, 00161, Italy

## Abstract

Retinitis pigmentosa (RP) comprises a group of inherited pathologies characterized by progressive photoreceptor degeneration. In rodent models of RP, expression of defective genes and retinal degeneration usually manifest during the first weeks of postnatal life, making it difficult to distinguish consequences of primary genetic defects from abnormalities in retinal development. Moreover, mouse eyes are small and not always adequate to test pharmacological and surgical treatments. An inducible paradigm of retinal degeneration potentially extensible to large animals is therefore desirable. Starting from the serendipitous observation that intraocular injections of a Rho GTPase activator, the bacterial toxin Cytotoxic Necrotizing Factor 1 (CNF1), lead to retinal degeneration, we implemented an inducible model recapitulating most of the key features of Retinitis Pigmentosa. The model also unmasks an intrinsic vulnerability of photoreceptors to the mechanism of CNF1 action, indicating still unexplored molecular pathways potentially leading to the death of these cells in inherited forms of retinal degeneration.

Retinitis pigmentosa (RP) includes a group of pathologies in which a mutation, typically in a retinal-specific gene, leads to primary rod photoreceptor loss, causing night blindness and reduction of the peripheral visual field^1^. This process is followed by the secondary death of cones, with consequent daytime blindness and loss of color vision and visual acuity up to near blindness. As a consequence of photoreceptor degeneration, inner retinal cells also undergo a process of regressive remodeling. RP is a rare disease, with an estimated incidence of 1/3,000-1/5,000 and the first symptoms usually appear during adolescence, with an overall progression that may take several years, mainly depending on the underlying mutation; blindness is anyway the inevitable outcome, with no cure yet^2^,^3^. Various animal models of RP (either naturally occurring or resulting from genetic manipulations) are available and actively exploited to study the progression of the pathology, to identify the mechanisms of retinal degeneration and to develop therapeutic strategies^4^,^5^,^6^. Some differences exist between human RP and rodent models of retinal degeneration; for instance, the progression of photoreceptor loss takes place in a periphery-to-center gradient in humans and it is opposite (i.e. center-to-periphery) in rodents. This reflects the fact that in humans rods predominate in the periphery of the retina, whereas in rodents (which miss a fovea and have very few cones) these cells reach the highest density in the central retina.

More importantly, in most of the rodent models of the disease, photoreceptor degeneration starts when retinal development is still ongoing, unlike what observed for human RP, which typically develops in young adulthood. Actually, in rodents, photoreceptor-specific genes become expressed typically during the second week of postnatal life, when retinal cells are still dividing and synaptic connections actively forming. Thus, genetic models of RP are limited by the fact that some of the effects on retinal morphology and function could be caused by incorrect or incomplete retinal development rather than by the process of retinal degeneration *per se*^7^. Human typical RP is not a developmental disease and therefore most animal models do not mimic faithfully the human condition^8^,^9^.

Adequate models of RP, able to mimic the pathology more closely to the human counterpart (albeit on a faster temporal scale) are necessary to understand the neurobiology of this disease and test potential therapeutic strategies.

Few inducible mouse models of RP have been developed, which however are limited by the non-tissue specific expression of the underlying mutation^10^,^11^,^12^.

Other inducible models have been generated using pharmacological agents such as iodoacetic acid (IAA), N-methyl-N-Nitrosurea (MNU) or Sodium Iodate^13^,^14^,^15^. The limit of this drug-based approach is that, rather than a given cell type, all retinal cells are targeted, so that the effect might be aspecific. This is very different from natural mutations responsible for RP, which are usually highly selective for both cell types and mechanisms of action. Moreover, a general toxicity is reported when the drugs are supplied by intravenous or intraperitoneal delivery^16^.

In this study we generated an inducible model of retinal degeneration that bypasses the window of retinal development. We exploited the bacterial toxin Cytotoxic Necrotizing Factor 1 (CNF1) to induce a primary, photoreceptor-specific damage in adult rats.

CNF1 is a protein toxin of approximately 110-kDa from pathogenic strains of *Escherichia coli* that acts as a modulator of the family of Rho GTPases (encompassing Rho, Rac and Cdc42 subfamilies), inducing actin reorganization in stress and retraction fibers^17^. CNF1 acts by deamidating a specific glutamine residue (glutamine 63 in RhoA or its homolog in other Rho proteins)^18^ to glutamate, leading to the inhibition of the intrinsic (and GAP-stimulated) GTPase activity. Therefore, GTP hydrolysis is blocked, with a persistent effector activation that has been detected up to several weeks following one single CNF1 brain injection^19^. Subsequent to deamidation by CNF1, cellular Rac1 is ubiquitinated (and subsequently degraded) by a proteasome-dependent mechanism^20^,^21^. Thus, the net result of CNF1 treatment depends on the balance between activation and degradation of its effectors^22^,^23^. A similar toxin, Cytotoxic Necrotizing Factor Y (CNFY), is produced by entheropathogenic strains of *Yersinia pseudotuberculosis*. Contrary to CNF1, CNFY is a selective activator of RhoA, RhoB and RhoC, and can be used as a powerful tool for constitutive RhoA activation without concomitant activation of Rac1 or Cdc42^20^,^24^.

Here we found that a single intravitreal injection of CNF1 was sufficient to induce a primary photoreceptor damage, resulting in a retinal degeneration model that recapitulates some of the main features of RP, although cone loss was observed to occur concomitantly to the degeneration of rods.

## Results

### Induction of retinal degeneration with a single CNF1 intraocular injection

We tested the effects of different CNF1 doses injected intraocularly in Long Evans rats to set up optimal conditions in which retinal photoreceptors (PR) are the primary target of the toxin.

Escalating doses (0.1, 1, 3 and 10 nM) of the toxin were injected in rat eyes and the effects assessed 14 days after injections. An evident dose-dependent effect of the molecule could be observed, with progressively more severe patterns of retinal degeneration at higher concentrations, resulting in major retinal cell degeneration and loss of laminar structure at 10 nM (see Supplementary Fig. S1).

While doses of the toxin 3 nM or higher induced nonspecific degeneration of all retinal cell types, lower doses produced selective abnormalities in the photoreceptor layer, indicating that these cells are the most sensitive to dysregulation of Rho GTPase signaling in the retina. Because a moderate and progressive pattern of retinal degeneration was desirable, for possible use in rescue studies, we choose to employ 2 nM CNF1 for further experiments. Afterwards, we used morphological, biochemical and functional tools to analyze systematically the obtained paradigm of retinal degeneration.

### Histopathological changes in CNF1-treated retinas

Three time points (i.e. 48 h, 7 days and 14 days post injection), corresponding to early, intermediate and late stages of retinal degeneration, were chosen to analyze CNF1-treated eyes, and evaluate the ability of CNF1 treatment to reproduce typical features of retinal degeneration of genetic origin.

#### Outer retina

**Figure 1:**
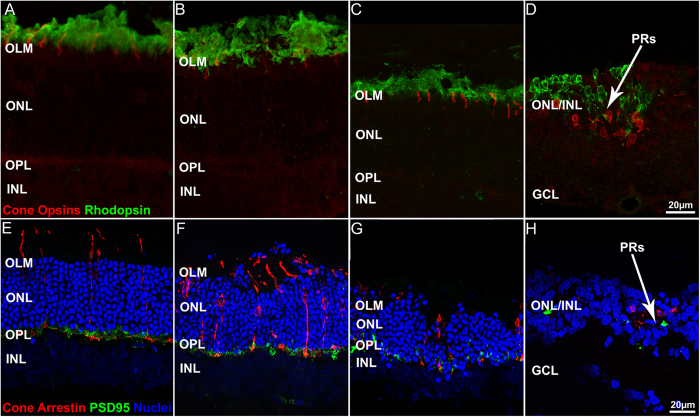
Morphological effects of CNF1 on the outer retina. Upper row: rod outer segments are stained in green with rhodopsin, and cones outer segment are stained in red by S and M/L wavelength cone opsin antibodies. Bottom row: cones are stained in red with cone arrestin, and photoreceptor terminals in green with PSD95. Nuclei are stained in blue with Hoechst dye. (**A**,**E**) control retinas. (**B**–**D**,**F**–**H**) CNF1 treated retinas harvested 48 hr (**B**,**F**), 7 days (**C**,**G**) and 14 days (**D**,**H**) post injection. Note the irregular course of the outer retina at early stages (**B**,**F**) and the uneven reduction of the ONL (**G**). Retinal organization appears profoundly altered at 14 days (**D**,**H**) with remnant photoreceptors dispersed in the INL and clustered in rosettes (arrows in **D**,**H**). Abbreviations here and in the following pictures: ONL, OPL: outer nuclear/plexiform layer. INL: inner nuclear layer; GCL: ganglion cell layer; OLM: outer limiting membrane; PRs: photoreceptors.

**Figure 2:**
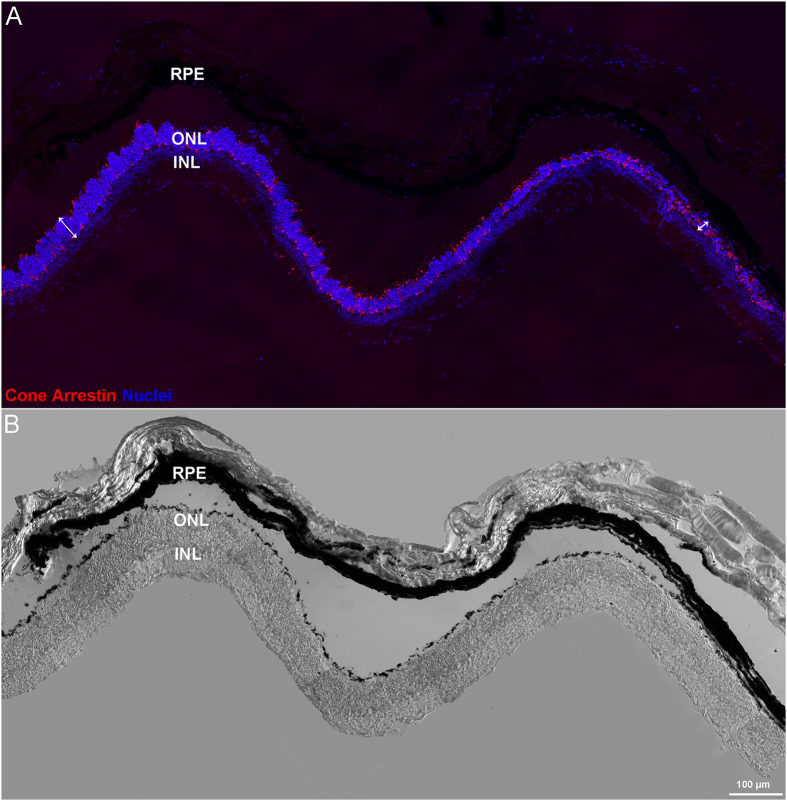
Centre to periphery retinal degeneration 7 days after toxin injection. Panels (A,B) show the same area of a 7 days post CNF1 injection retina, viewed in fluorescence (**A**) and in bright field illumination with Nomarski optics (**B**). In A, nuclei are stained blue with Hoechst dye and cones in red with cone-arrestin antibodies. Double headed arrows in A highlight the different width of the ONL in peripheral (left) and central (right) retinal regions.

Early effects of CNF1 are primarily visible in the outer retina, where a characteristic undulation of the outer limiting membrane is observed as early as 48 h after injection (see Fig. 1, panels A, B and F). Photoreceptors begin to die as shown by the appearance of pycnotic nuclei (indicative of DNA condensation), and the outer nuclear layer (ONL) starts to lose its regular organization. A reduction of the ONL thickness (from 10 to 5 rows of nuclei in the center) is visible 7 days after treatment (see Fig. 1, panels C and G). Fourteen days after treatment, only few photoreceptors remain; some of them appear clustered in typical rosettes, in which surviving cells become organized circularly around a central core. They are surrounded by remnant, displaced inner retinal neurons. The outer plexiform layer gradually disappears and the laminar organization of the retina is irreversibly lost (see Fig. 1, panels D and H). Retinal degeneration progresses from the center to the periphery, coherently with genetic rodent models of the pathology (see Fig. 2, and Supplementary Figs S3 and S4, panels A and C).

#### Inner retina

**Figure 3:**
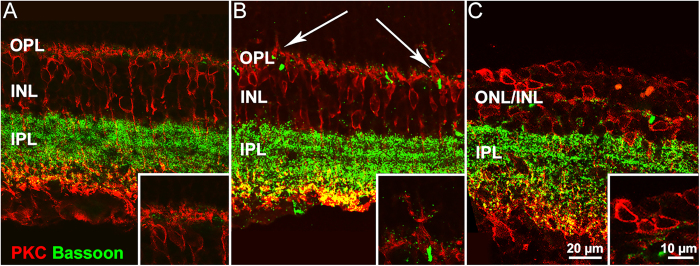
Morphological effects of CNF1 in the inner retina. Rod bipolar cells are stained by PKC antibodies (red signal); presynaptic glutamatergic terminals are stained by Bassoon (green signal). (**A**) Control retina. (**B**,**C**) CNF1 treated retinas. Remodeling of bipolar cells is visible beginning 7 days after treatment, in the form of dendritic sprouting (arrows in B and inset). 14 days after toxin injection, the dendrites of rod bipolar cells have completely retracted (**C**). Because of photoreceptors have died out (inset), Bassoon staining in their presynaptic terminals in the OPL is not detectable at this stage.

**Figure 4:**
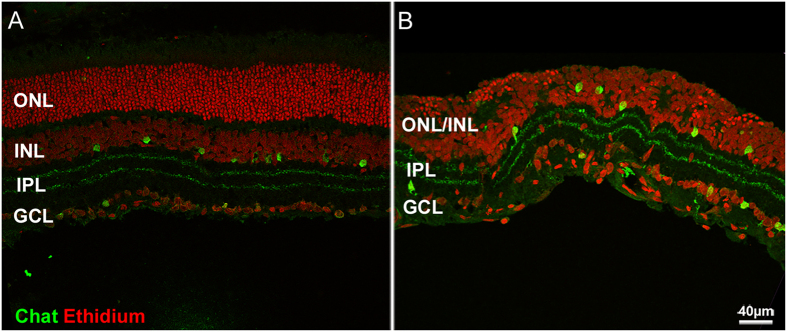
Retinal degeneration following CNF1 administration proceeds from the outer to the inner retina. Red: ethidium nuclear staining. Green: ChAT staining of cholinergic amacrine cells. (**A**) Control retina. (**B**) CNF1-injected retina, 14 days after injection. The distinction between ONL and INL has been lost, and yet the cholinergic bands are still clearly visible in B. Sparing of the innermost retina is typical of the RP phenotype.

Following the initial phase of photoreceptor death and accompanying the final stages of ONL progressive thinning, inner retinal neurons undergo phases of reactive and regressive remodeling, although no reduction of INL thickness is observed (see Supplementary Fig. S3, panel B). Specifically, 7 days after injection, dendritic sprouting of bipolar cell is clearly visible (see Fig. 3, panel B). Sprouts are short, dendritic branches pointing toward the outer retina. However, with the progression of retinal degeneration, second order neurons (both rod bipolar and horizontal cells) retract their dendrites while their cell bodies become misplaced between the INL and those of remnant photoreceptors (see Fig. 3, panel C). Cholinergic (ChAT) bands, formed by the dendrites of starburst amacrine cells, remain normally positioned at 1/3 and 2/3 of the inner plexiform layer and maintain a parallel course even when the outer retina has totally degenerated. However, ChAT bands are abnormally wavy along the tangential plane (see Fig. 4).

Ganglion cells and the ganglion cell layer appear less affected by retinal degeneration, i.e. the general morphology of ganglion cells and their dendritic lamination in the IPL do not show major abnormalities.

#### Gliosis

**Figure 5:**
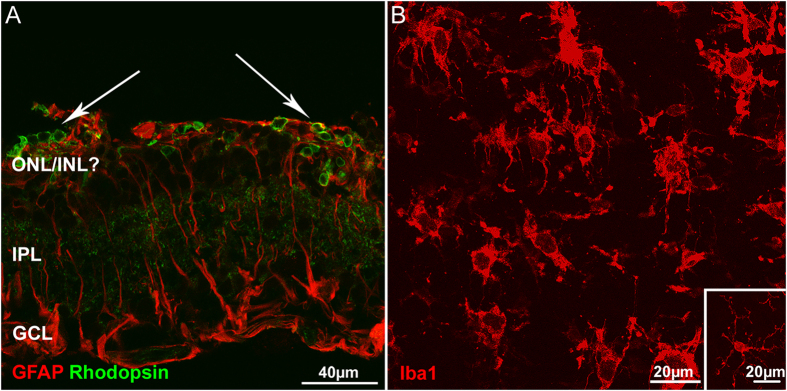
Activation of macro and microglia upon CNF1 injection. (**A**) Muller cells reactive gliosis 14 days post CNF1 injection. Red: GFAP; green: rhodopsin staining. Arrows point to clusters of residual photoreceptors (green). (**B**) Retinal whole mount stained with Iba1 antibody against microglia. Cells are in the typical amoeboid, active shape. The focal plane is in the outer retina. The inset shows an example of a quiescent, ramified microglial cell from a control retina.

CNF1 injections cause a robust activation of retinal glial cells. Müller cells show increased reactivity for GFAP all over the retinal surface and increased thickness of the radial processes. Microglia also switches from the quiescent, ramified morphology, to the amoeboid shape typical of the phagocytic status (see Fig. 5, panel B). Activated microglial cells are found mainly in the outer retina and are evidently committed to engulf the nearby dying photoreceptors. This observation confirms that the latter are the main cell population undergoing cell death upon CNF1 injection.

Because of extensive retinal morphological rearrangements, we investigated whether CNF1 treatment induced mitosis in retinal and/or in microglial cells using an antibody specific for the cell division marker Histone 3 phosphate. No active mitosis was found in CNF1 treated (or in control) retinas; the retina from a 1-day old rat used as positive control (see Supplementary Fig. S5).

### Functional analysis of CNF1-induced retinal degeneration

CNF1 reduces both scotopic and photopic electroretinogram (ERG) responses.

**Figure 6:**
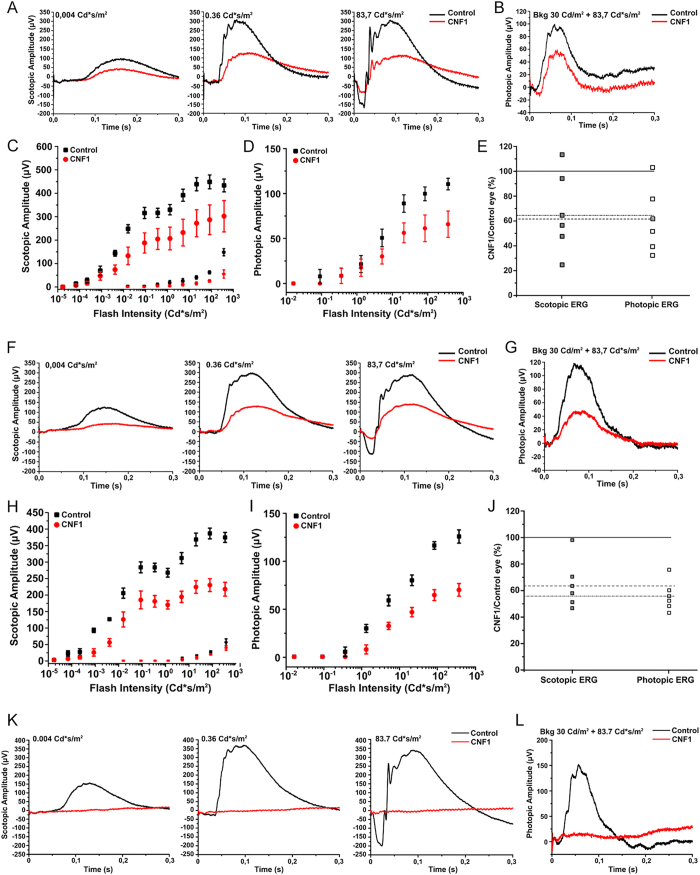
Functional effects of CNF1 toxin investigated by ERG recordings 48 h and 14 days after injection. (**A**,**B**) Representative example of scotopic and photopic ERG recordings (black trace: control eye, red trace: CNF1 injected eye) after 48 h. (**C**,**D**) Sensitivity curves (the amplitude as function of increasing intensity of light) of a-wave and b-wave from scotopic (**C**) and photopic (**D**) ERG. (**E**) Percentage residual response (compared to control eye) of scotopic and photopic ERG response (solid line = average control response, dotted line = average scotopic response, dashed line = average photopic response). (**F**,**G**) Representative example of scotopic and photopic ERG recordings after 7 days. (**H**,**I**) Sensitivity curves of a-wave and b-wave from scotopic (**H**) and photopic (**I**) ERG. (**J**) Percentage residual response (compared to control eye) of scotopic and photopic ERG response. (**K**,**L**) Representative example of scotopic and photopic ERG recordings after 14 days.

ERG recordings in CNF1 injected and control eyes clearly demonstrated the functional consequences predictable on the basis of the morphological analysis, and namely a reduction in the amplitude of both scotopic and photopic responses as early as 48 h after CNF 1 treatment (Fig. 6A–E).

The amplitude of the ERG recorded from the eyes treated with the toxin appeared further reduced at the subsequent time point examined (7 days after injection, Fig. 6F–J) and the ERG was completely suppressed 14 days after treatment (Fig. 6K,L). Figure 6E–J shows how the amplitude of both scotopic and photopic ERGs decreases in time with a similar time course both at 48 h and 7 days, indicating that the toxin affects similarly rods and cones. Furthermore, the ratio of scotopic b/a wave measured at 48 h and 7 days in control and in treated animals is not significantly changed, suggesting that the target of the toxin is mainly directed to photoreceptors rather than to second order neurons (see Supplementary Table S4).

### Rac/Cdc42 are involved in CNF1-induced retinal degeneration

**Figure 7:**
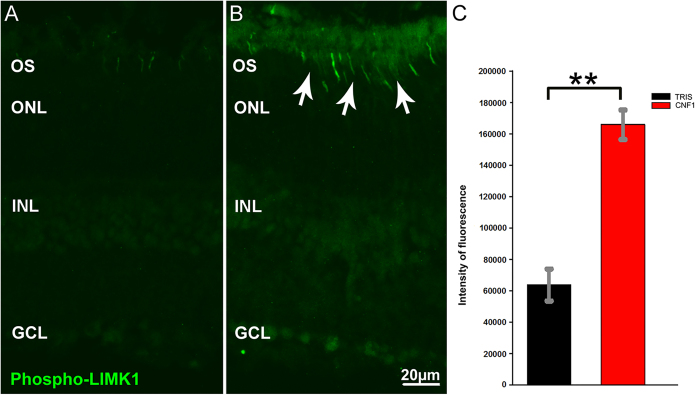
Activation of Rac1 and Cdc42 downstream effector. Immunocytochemistry for phosphorylated (Thr508) LIM Kinase1 (green), 48 hrs post injection of vehicle (**A**) or CNF1 (**B**). Quantification of fluorescence shows that the intensity of staining in toxin injected retinas is 2.6 times brighter with respect to control preparations in the layer of photoreceptor outer segments (OS). (**C**) Diagram of mean values of fluorescence intensity in CNF1 (red) and control (black) preparations. Error bars represent s.e. of the mean.

To demonstrate activation of Rho GTPase-dependent signaling pathways in CNF1-treated retinas, we assessed phosphorylation of LIMK1, one of the key steps in Rho- and Rac-dependent reorganization of the actin cytoskeleton. Indeed, two downstream effectors of Rho GTPases, P21 Protein (Cdc42/Rac)-Activated Kinase (PAK1) and Rho-associated protein kinases (ROCK), activate LIMK1 by phosphorylation at Thr-508. We therefore assessed the distribution of phosphorylated LIMK1 in control and CNF1-treated retinas at 48 h (see Fig. 7, panel B) using immunocytochemistry. We found robust labelling for activated LIMK1 in the rod outer segments in CNF1-injected, but not control, samples (see Fig. 7, panel A). Relative measurements of fluorescence intensity in the layer of photoreceptor outer segments show statistically significant increase of PLIMK1 signal in CNF1 treated retinas (Two-tailed Student T test. P = 0.0036).

It should be noticed that activation of LIM kinase 1 is visible even in retinal regions where, 48 hours after injection, the undulation of the outer limiting membrane (OLM) is still minimal or absent. This suggests that the activation of Rac1 and Cdc42 pathways precedes retinal degeneration.

To narrow the search of possible molecular effectors of the retinal degeneration induced by CNF1, we took advantage of *Yersinia pseudotubercolosis* Cytotoxic Necrotizing Factor (CNFY), a toxin that selectively activates RhoA, but not Rac/Cdc42^24^. The toxin was injected under the same conditions used for CNF1, and treated and control eyes were collected after 14 days.

CNFY did not cause any adverse effect on the morphology of the retina, which remained virtually unchanged in its fine structure and lamination (Supplementary Fig. S7). These data suggest that the observed effects of CNF1 on the retina depend upon the activation of Rac1 and/or Cdc42 GTPases, while a role of RhoA can be excluded.

### CNF1 induces oxidative stress in retina

It is known that Rac1 is a component of NADPH oxidase that produces reactive oxygen species (ROS) in non-phagocytic cells. Indeed, it has been shown that its inactivation in rod photoreceptors makes these cells more resistant to photo-oxidative damage^25^.

We hypothesized that Rac1 constitutive activation following CNF1 treatment induced and increase in ROS and in retinal oxidative stress, contributing to primary photoreceptor death in our inducible model of retinal degeneration.

**Figure 8:**
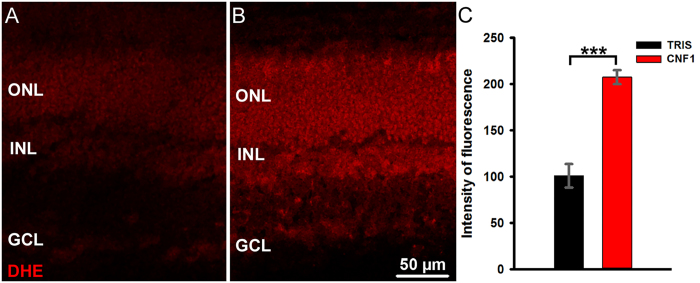
DHE assay on retinal sections 24 hours after vehicle (**A**) or CNF1 (**B**) intraocular injections. In the presence of ROS, DHE is converted to ethidium, which stains DNA, producing a red fluorescence signal. Quantification of fluorescence shows that the intensity of staining in toxin-injected retinas is twice as bright with respect to control preparations (**C**). Bars represent mean values of fluorescence intensity; error bars represent s.e. of the mean.

We found that retinas treated with CNF1 do exhibit a more intense dihydroethidium (DHE) staining compared to the opposite, control retinas (see Fig. 8). The intensity of fluorescence DHE staining found in CNF1 injected retinas is, on average, twice as bright as controls, indicating the presence of higher amounts of ROS in the toxin-exposed preparations (Two-tailed Student t test. P = 0.000609).

In summary, a single, 2 nM injection of CNF1 in the adult rat eye leads to a progressive retinal degeneration that starts from the photoreceptor layer and progresses in time toward the inner retina. This paradigm of retinal decay is associated to increased levels of ROS, appears independent from the activation of RhoA but is linked to Rac1 and Cdc42 activation, and is paralleled by profound abnormalities of the retinal ERG, which progressively becomes extinct.

## Discussion

We successfully implemented a paradigm of retinal degeneration that summarizes key features of typical retinitis pigmentosa (RP), and specifically:

-Retinal degeneration can be induced in young adult animals, similarly to what observed in humans, for which the typical age of RP manifestation is late adolescence, outside the window of retinal development;
-Photoreceptors are the cells primarily affected. These cells undergo a process of progressive death;
-A center to periphery gradient in the onset of retinal morphological abnormalities is clearly observed;
-A close correspondence of anatomical and electrophysiological (ERG) findings is found;
-ERG data indicate the toxin effects are mainly on photoreceptors, albeit without a preference for rods or cones.
-Typical remodeling (sprouting, dendritic atrophy, cell body misplacement etc.) of second order neurons (with a sparing of innermost retinal cells) is observed following photoreceptor death;
-The severity of the retinal degeneration depends strictly on the dose of toxin used and progresses over time for a given dose.

We noted that the undulation of the outer limiting membrane and photoreceptor death began 2 days after injection, while sprouting of second order neurons was detected 7 days after treatment. These data suggest that remodeling of the inner retina is a secondary effect of the death of photoreceptors (like in RP), rather than caused by a direct action of the toxin on second order neurons. This interpretation is supported by the early and robust activation of LIMK1 in rod outer segments (but not in the inner retina) following CNF1 treatment (see Fig. 7).

Because we could demonstrate that CNF1 treated retinas showed higher levels of ROS compared to controls, we suggest that a possible mechanism of action of CNF1 on the retina involves the constitutive activation of Rac1, which in non-phagocytic cells is part of the enzymatic complex of NADPH oxidase. This would lead to the observed increment of oxidative byproducts. This could also explain why the primary effects of the toxin are detected selectively in photoreceptors: these cells are known to be highly vulnerable to photo-oxidative stress. This is in line with the finding that depletion of Rac1 in mouse rods makes them more resistant to photo-oxidative damage^25^ and that persistent activation of Rac1 obtained with transgenesis leads to retinal degeneration in mice^26^,^27^. It is important to note that CNF1 action depends on the balance between activation and degradation, and that CNF1-activated Rac1 is known to be eliminated by a proteasome-dependent mechanism. Thus, we cannot exclude that the progression of degeneration at late stages is paradoxically caused by insufficient levels of Rho GTPases.

Contrary to Rac1 depletion studies, experiments performed to decrease the expression of Cdc42 from the retina have led to very different results. Cdc42 is necessary for the proper development of the retina^28^, while its removal in adult animals has neither adverse effects, nor exerts a protective action toward light induced retinal degeneration^29^. We found that a selective RhoA activation is not able, by itself, to induce retinal degeneration, as retinas treated with CNFY do not show any abnormality. Altogether, these data suggest that Rac1 activation in photoreceptors is the primary event leading to RP-like retinal reorganization following CNF1 treatment.

CNF1 has been used experimentally to manipulate adult neurons as well as to ameliorate the outcome of CNS pathologies, such as Rett syndrome and Alzheimer Disease (AD), because of its ability to promote neuronal plasticity and to protect astrocytes^22^,^30^.

It might seem surprising that the same toxin could exert beneficial effects on some of the abnormalities found in Rett syndrome or AD, while exerting degenerative effects on photoreceptors. However, in the case of Rett syndrome, CNF1 was used to regulate cytoskeleton assembly of dendritic spines in a system in which a physiological Rho GTPase activity was defective^30^. Conversely, in the case of CNF1 eye injections, we observe that the toxin impacts on photoreceptors, which have high metabolic rates and continuous membrane turnover and are therefore highly sensitive to oxidation and cytoskeletal changes. CNF1 preferential action on photoreceptors may be due to either uptake of the bacterial toxin, which occurs via receptor-mediated endocytosis, or cell-specific sensitivity to Rho GTPase perturbation. Two CNF1 receptors, laminin receptor^31^ and Lutheran adhesion glycoprotein/basal cell adhesion molecule (Lu/BCAM)^32^ have been recently identified but it is not known whether they are predominantly expressed in photoreceptors. Specificity of CNF1 action in photoreceptors may also result from a selective vulnerability: these metabolically very active cells are likely sensitive to dysregulation of intracellular signaling, so that continuous activation of Rho GTPase signaling and changes in cytoskeletal organization may lead to their degeneration. This interpretation is supported by the cytotoxic effects of CNF1 on proliferating cells such as glioma cells^33^. In addition, mutations in proteins involved in the dynamics of actin cytoskeleton lead to severe photoreceptor phenotypes and retinal blindness, as it is the case of ciliopathies including Usher syndrome^34^.

Altogether, we believe that the specific, toxin-inducible model of retinal degeneration described here offers several advantages, including the possibility to be exported to mammals of larger size, like rabbits and pigs. These are reference species for pharmaceutical therapeutic approaches in ophthalmology. In perspective, CNF1-induced retinal degeneration could be also implemented in monkeys (which otherwise do not develop RP), a model system for testing therapies close to human translation. Noticeably, rats treated with CNF1 did not show damages to other organs or general behavioral abnormalities, unlike drug-induced models described in other studies^16^. Indeed, albeit Rho GTPases are ubiquitously expressed in cells, the CNF1 dosage used here appears to target primarily photoreceptors. Higher doses do affect all retinal layers simultaneously, as shown in Supplementary Fig. S1.

Because CNF1 induces a rapid and quite specific process of photoreceptor death, it could be useful in cell transplantation studies to eliminate residual, abnormal photoreceptors, favoring the implant of healthy precursors. This process would also be favored by the observation that CNF1 decreases the stability of the outer limiting membrane, a significant physical barrier to the migration and integration of precursors in the adult host retina^35^.

Finally, mechanistic studies on CNF1 toxin might reveal unknown pathways existing in normal photoreceptors that are susceptible to degeneration of these cells if altered or mutated. For example, a link has been established between NADPH oxidase and progressive retinal degeneration^36^,^37^ while a direct connection has been shown between Rac1 activation and retinal degeneration in mouse models^38^.

CNF1 signaling is composed by various molecular effectors. Clearly, photoreceptors are highly sensitive to these molecules whose balance might affect their survival. It is possible that mutations of this molecular machinery are responsible for forms of RP for which the parent mutation has not been identified yet. This study suggests searching for candidate disease genes among molecules belonging to the CNF1 signaling cascade^36^, hopefully contributing to the still incomplete catalogue of genes for inherited retinal diseases (see https://sph.uth.edu/retnet/sum-dis.htm).

## Methods

### Animals

All the experimental procedures were in accordance with European and Italian laws regulating the experimental use of animals for research. All experimental protocols were approved by the Italian Ministry of Health and by the CNR Neuroscience Institute Animal Care and Use Committee (Protocol #Strettoi14D). Adults Long Evans Hooded rats (age 4–12 months) were used in this study. A total of 24 rats were used to set the toxin dosage able to induce a retinal degeneration mimicking an RP like phenotype. A dosage of 2 nM CNF1 was chosen for immunohistochemistry assays and photopic ERG recordings, then using a total of additional 34 rats (19 for morphological experiments and 15 for ERG recordings, respectively).

### CNF1 ocular injections

CNF1 was purified as described previously^29^. For intraocular injections, the toxin was diluted in Tris HCl 1 M pH 7.4 from the initial concentration of 870 nM. After dilution, the toxin was stored at 4 °C and used within a month.

For intraocular injections, individual rats were pre-anesthetized with a gaseous mixture of 3–4% isoflurane in oxygen. The rats were then moved under a surgical microscope where the anesthesia, channeled through a mask, was maintained with a mixture of isoflurane in oxygen at 2.5%. Intraocular injections were performed in the vitreous body, using a glass capillary filled with the toxin and hand driven by an oil micromanipulator. A volume of 2 μl was injected at the *ora serrata*. Control injections consisted of equal volumes of vehicle only, or of boiled volumes of the toxin. Gaseous anesthesia allows recovery of the animals within minutes and the animals were returned to their cages after the injections.

### Retina explants

Rats were deeply anesthetized with intraperitoneal injections of avertin (6 gr of 2,2,2 tribromoethanole in 1% tert-amyl alcohol) at dose of 10 ml/Kg of body weight.

Eyes were rapidly enucleated, marking the dorsal pole with a skin marker, and immersion-fixed in 4% paraformaldehyde (PFA) in 0.1 M phosphate buffer (PB) for 30 minutes. The animals were sacrificed with an overdose of the same anesthetic. The eyes were then dissected to remove lens and cornea, thus obtaining eye-cups. A reference cut was made in correspondence of the dorsal pole, both in the sclera and the retina.

Eyecups were post-fixed for 30 minutes in the same fixative, then washed extensively in PB, infiltrated with 30% sucrose in the same buffer, followed by OCT embedding medium (Sakura, Tissue-Tek^®^ O.C.T™ cat #4583). Finally, the eyecups were frozen in cold isopenthane and serially sectioned at a cryostat in 14 μm thick sections.

The eyes used for dihydroethium (DHE) assay were rapidly enucleated, embedded in OCT and immediately frozen in cold isopenthane, without fixation.

### Fluorescence Immunocytochemistry (ICCH)

To study the morphology and survival rates of retinal cells in preparations from treated and control rats, we used ICCH with cell-type specific antibodies followed by confocal microscopy.

Cryostat retinal sections were incubated in blocking solution containing 0,3% Triton X-100, 5% goat or donkey serum, 0.01 M PBS (pH 7.4), at RT °C for 2 hours. Primary antibodies were diluted in the same buffer with 0,1% Triton X-100, 1% goat or donkey serum and revealed with anti-mouse/anti-rabbit Alexa Fluor 488 or anti-mouse/anti-rabbit Rhodamine RedX-conjugated secondary antibodies (all diluted 1:1,000), (Jackson Immunoresearch laboratories, USA). Retinal sections were counterstained with ethidium homodimer 1, or with YOYO-1 or with Hoechst (all diluted 1:1,000) to reveal nuclei (Invitrogen, Life Technologies).

For rod and cone identification, antibodies against rhodopsin (Sigma, O4886), cone opsins (Merk-Millipore, AB5407 and AB5405) and cone arrestin (Merk-Millipore, AB15282) were used. To study the morphology of inner retinal neurons and synapses, antibodies against PKC alpha (Sigma, P4334 or P5704), Smi32 (Covance, Smi32R), Bassoon (Enzo Life Science, ADI-VAM-PS003), Ribeye (BD Biosciences, 612044), PSD95 (AbCam Ab13552), Chat (Merck-Millipore, AB144P) and anti synaptotagmin 2 (Znp1) (Zebrafish International Resource Center, USA) were used. Glial activation was assessed with antibodies against GFAP (Sigma, G9269) and Iba1 (Wako, 019–19741). Mitotic activity was analyzed with an antibody marker of cell division, Phospho Histone H3 (Merk-Millipore, 06–570). Activation of Lim Kinase 1 was assessed with an antibody recognizing its phosphorylated form at threonine 508 (Sigma Aldrich, SAB4300103). Retinal sections and whole mount retinas were examined with a Zeiss Axio Plan microscope and acquired in brightfield with a Zeiss AxioCam camera or with a Zeiss Imager Z2 fluorescence microscope equipped with an ApoTome 2 system. Systematic imaging was also obtained with a Leica TCS-SP confocal microscope. Acquisition parameters (width along the z-axes, laser intensity, photomultiplier gain and pinhole size) were kept constant for all the samples examined.

### Nuclear layers measurements

Retinal sections from control and CNF1 injected eyes at different time points (48 h, 7day and 14 days after injection) were stained with micromolar solution of Hoechst nuclear dye (Invitrogen, Life Technologies) and imaged with a Zeiss Axioplan epifluorescence microscope equipped with a Zeiss Axio Cam camera and a 20x objective. Sections from 3 different eyes (from 3 different CNF1 injected and control animals) were used for each time point; 3 near-equatorial sections per retina were measured, sampling both central and peripheral eccentricities.

Measures of outer and inner nuclear layer thickness were made on sections images with MetaMorph program; the statistical analysis, t-test, was performed with SigmaPlot 12.

### Histochemistry: Dihydroethidium (DHE) assay

To detect retinal oxidation, we used a histochemical assay based on Dihydroethidium (Sigma Aldrich, Cat #37291), a reduced form of ethidium homodimer. In the presence of ROS, DHE is oxidized to ethidium with specific DNA intercalant ability and red fluorescence.

Dihydroethidium powder was resuspended in dimethilsulfoxide at 10^−2^ M and stored at −20 °C. As dihydroethidium could oxidize in the presence of light, all the steps of the assay were conducted in the dark. Cryostat sections from both CNF1 and vehicle treated eyes were mounted on each glass slide to minimize experimental variability. Sections were washed 3 × 10 minutes with PBS and incubated with DHE, diluted in PBS to final concentration of 10^−6^ M, at 37 °C for 30 minutes. Afterwards, the sections were washed 3 × 10 minutes in PBS, mounted and immediately examined by confocal microscopy.

As for ICCH, acquisition parameters were kept constant for all the samples examined.

### Quantification of fluorescence and statistical analysis

Confocal images obtained from phospho-LimK1 and DHE experiments from vehicle and CNF1 treated retinas were analyzed with a specific routine of MetaMorph program for fluorescence quantification. Measures obtained from images of 10 sections for each experimental group were statistically compared using Sigma Plot 12 software. For each experimental group, a normality test was run, followed by two tailed t-test (alpha = 0.05).

### Electroretinogram (ERG)

Rats were dark-adapted overnight and then anesthetized with an intraperitoneal injection of 20% urethane (1 ml/kg body weight), mounted in stereotaxic position and setup for ERG recordings^39^. Gold extra-corneal electrodes were used, while the ground electrode was a gold plate placed in the animal mouth. Body temperature was maintained at 37 °C. Pupils were dilated by administration of tropicamide (1%) eye drops, and the cornea was kept moist with a methylcellulose solution. An electronic flash unit generated a light stimulus of 492 nm whose energy decayed with a τ of 1.7 ms. Full-field stimulation was achieved using a Ganzfeld sphere, and flash intensities were attenuated by neutral-density filters. Rats were subjected to thirteen different flash intensities for scotopic ERG and seven for photopic ERG, each repeated five times, with an interstimulus interval that ranged from 20 s for dim light to 1 min for the brightest flashes^40^. The flash luminance was measured at the corneal plane in photometric units (cd·s/m2) using a Minolta CS100 photometer with a scotopic filter. Responses were differentially amplified, band-pass-filtered at 0.3–500 Hz, digitized at 0.25- to 0.5-ms intervals, and stored on disk for processing. The amplitude of the a-wave was taken as the difference between baseline and the value after 7 ms from flash, whereas the amplitude of the b-wave was measured from the minimum value of the a-wave to the peak of the b-wave. Isolated cone components were obtained by superimposing test flashes on a background of saturating intensity for rods (30 cd/m2).

## Additional Information

**How to cite this article**: Guadagni, V. *et al.* The bacterial toxin CNF1 as a tool to induce retinal degeneration reminiscent of retinitis pigmentosa. *Sci. Rep.* **6**, 35919; doi: 10.1038/srep35919 (2016).

## Supplementary Material

Supplementary Information
