# Corrigendum: The bacterial toxin CNF1 as a tool to induce retinal degeneration reminiscent of retinitis pigmentosa

**DOI:** 10.1038/srep37882

**Published:** 2016-12-09

**Authors:** Viviana Guadagni, Chiara Cerri, Ilaria Piano, Elena Novelli, Claudia Gargini, Carla Fiorentini, Matteo Caleo, Enrica Strettoi

Scientific Reports 6: Article number: 3591910.1038/srep35919; published online: 10 24 2016; updated: 12 09 2016

In this Article the author contributions section is incomplete:

“V.G. planned experiments, performed experiments, analyzed data and wrote the manuscript; C.C. planned experiments, analyzed data and critically read the manuscript; C.G. planned and performed the ERG experiments, analyzed data and critically read the manuscript; E.N. provided technical support, analyzed data and critically read the manuscript; C.F. provided fundamental reagents and critically read the manuscript; M.C. planned experiments, analyzed data and critically edited the manuscript; E.S. planned experiments, analyzed data and wrote the manuscript”.

Should read:

“V.G. planned experiments, performed experiments, analyzed data and wrote the manuscript; C.C. planned experiments, analyzed data and critically read the manuscript; C.G. planned and performed the ERG experiments, analyzed data and critically read the manuscript; I.P. planned and performed the ERG experiments, analyzed data and critically read the manuscript; E.N. provided technical support, analyzed data and critically read the manuscript; C.F. provided fundamental reagents and critically read the manuscript; M.C. planned experiments, analyzed data and critically edited the manuscript; E.S. planned experiments, analyzed data and wrote the manuscript”.

