# Supplementary material for: The bacterial toxin CNF1 as a tool to induce retinal degeneration reminiscent of retinitis pigmentosa
Source: Sci Rep. 2016 Oct 24;6:35919. doi: 10.1038/srep35919 (PMC5075935; doi:10.1038/srep35919)
Supplement: Supplementary Information [file srep35919-s1.pdf]

Supplementary Information

The bacterial toxin CNF1 as a tool to induce retinal degeneration reminiscent of Retinitis Pigmentosa

Viviana Guadagni[1], Chiara Cerri[1,2], Ilaria Piano[3], Elena Novelli[1], Claudia Gargini[3], Carla Fiorentini[4], Matteo Caleo[1], Enrica Strettoi[1*].

1 Neuroscience Institute, Italian National Research Council (CNR), Pisa, 56124, ITALY

2 Accademia dei Lincei, Rome,00165, ITALY

3 Department of Pharmacy, University of Pisa, Pisa, 56100, ITALY

4 Istituto Superiore di Sanità, Rome, 00161, ITALY

***Corresponding Author. Istituto di Neuroscienze del CNR, Area della Ricerca, Via G. Moruzzi 1, 56124 Pisa, Italy**

enrica.strettoi@in.cnr.it

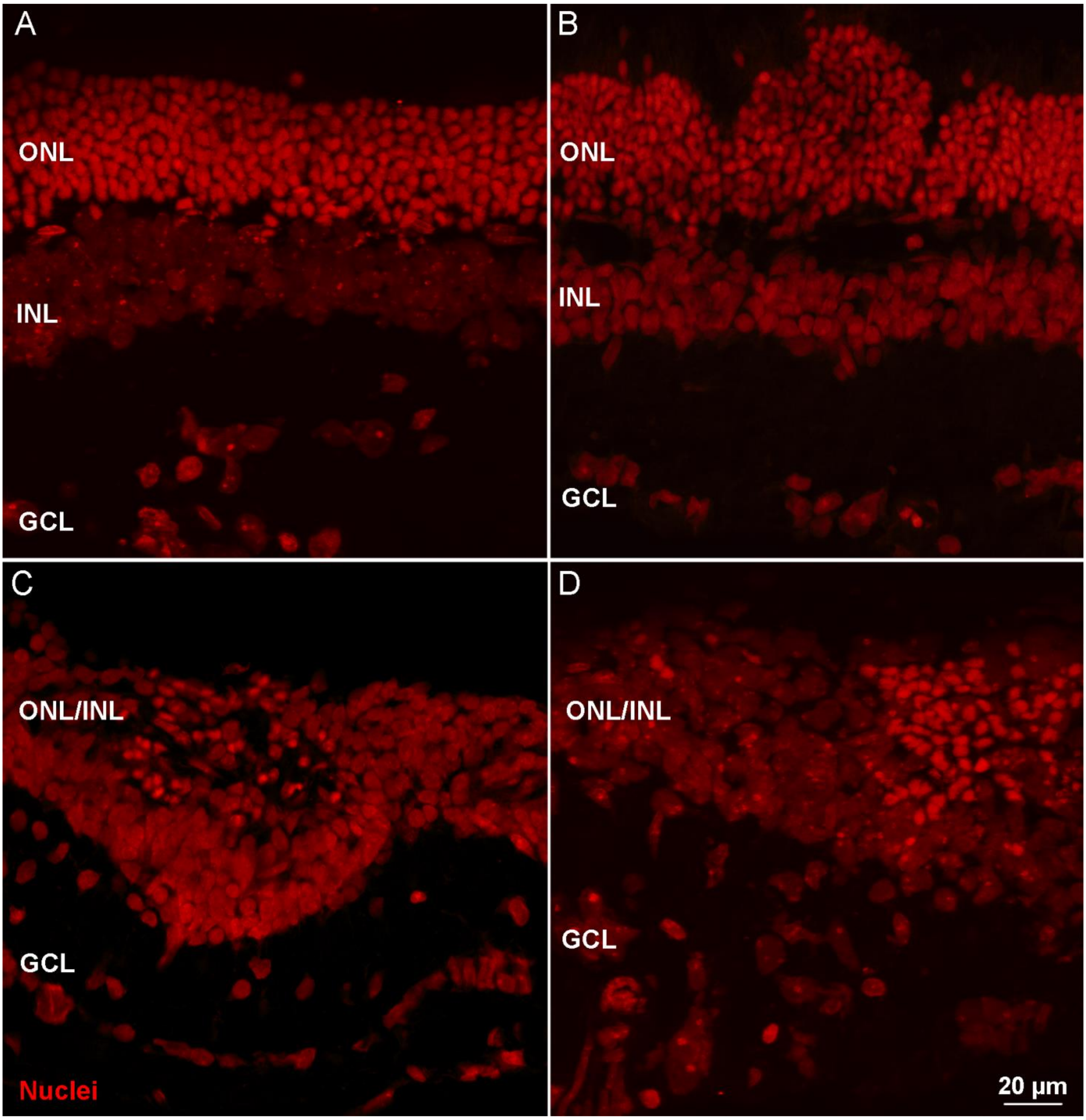

Supplementary Figure S1: Dose dependent CNF1 effects. Nuclear staining used to assess retinal morphology 14 days after injection of CNF1 at various doses; panels A-D: retinas treated with CNF1 at escalating concentrations, respectively 0.1, 1, 3 and 10 nM. Panels A and D are stained with ethidium, panel B and C with YOYO-1 but shown in fake red.

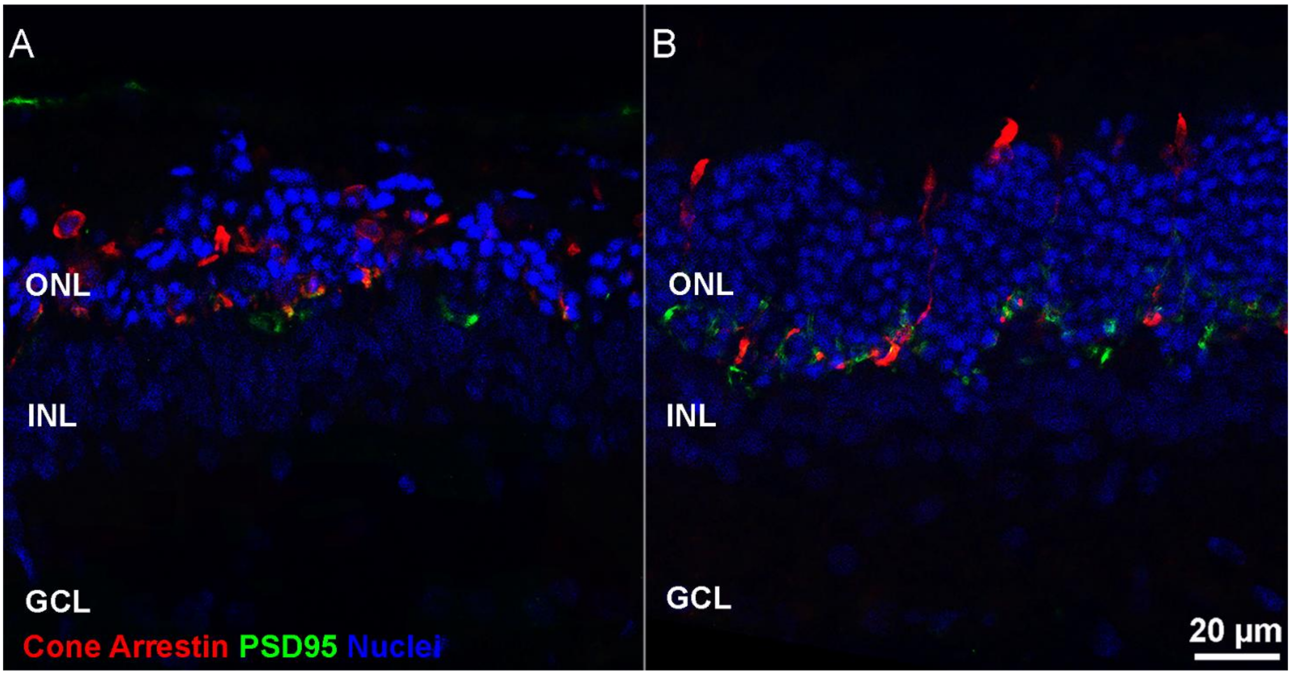

Supplementary Figure S2: Centre to periphery retinal degeneration 7 days after CNF1 toxin injection. Cones are stained in red with cone arrestin and photoreceptor terminals are stained in green with PSD95. Nuclei are stained in blu with Hoecst. Both panels are from same section. A: central retina, in the proximity of the optic nerve head. B: peripheral retina. In A, photoreceptor rows are only 3 or 4 , while they are close to 9 rows in B.   An undulatory pattern of the outer retina is widespread.

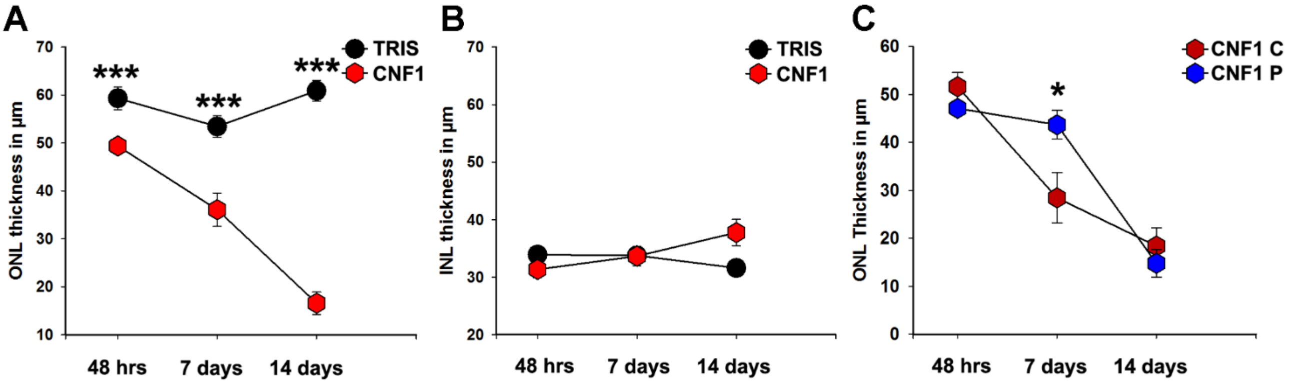

Supplementary Figure S3: Morphometry of retinal nuclear layers 48 hrs, 7 days and 14 days after injection of vehicle (black) or toxin (reds). A: The decrement of ONL thickness in time is very pronounced. B: the average thickness of INL does not decrease with time. C: ONL thickness in central (dark red dots) vs peripheral (blue dots) retinal areas in CNF1 injected samples. Seven days after injection, the ONL is thicker in retinal periphery. Hence, CNF1 induced retinal degeneration proceeds in a center to periphery gradient and mainly involves the photoreceptor layer.

flash	cd*s/m^2	b wave / a wave mean CTR 48hrs	SE	b wave / a wave mean CNTF1 48 hrs	SE	p
3	1.3	6.77218	0.66203	7.03795	1.71731	0.88875
2.4	5.12	3.91917	0.12773	4.63104	1.40538	0.62754
1.8	21.2	3.30949	0.12749	3.34956	0.6433	0.95277
1.2	83.7	2.91782	0.05982	2.75709	0.35935	0.67074
0.6	377	2.87713	0.10524	2.39595	0.2374	0.10101

flash	cd*s/m^2	b wave / a wave mean CTR 7d	SE	b wave / a wave mean CNTF1 7d	SE	p
3	1.3	8.86383	1.07563	7.92642	0.6104	0.47021
2.4	5.12	4.45849	0.21231	4.84269	0.64758	0.58837
1.8	21.2	3.28002	0.13197	3.74281	0.38514	0.28854
1.2	83.7	2.94136	0.12047	3.27693	0.23215	0.23541
0.6	377	2.61449	0.11269	2.90189	0.13393	0.13922

Supplementary Table S4. b wave to a wave ratio in scotopic conditions, 48 hr and 7 days after injection. ERG recordings show no differences between control and CNF1 eyes at both time points. These results show that the rods to cones ratio is the same in all the exhamined conditions.

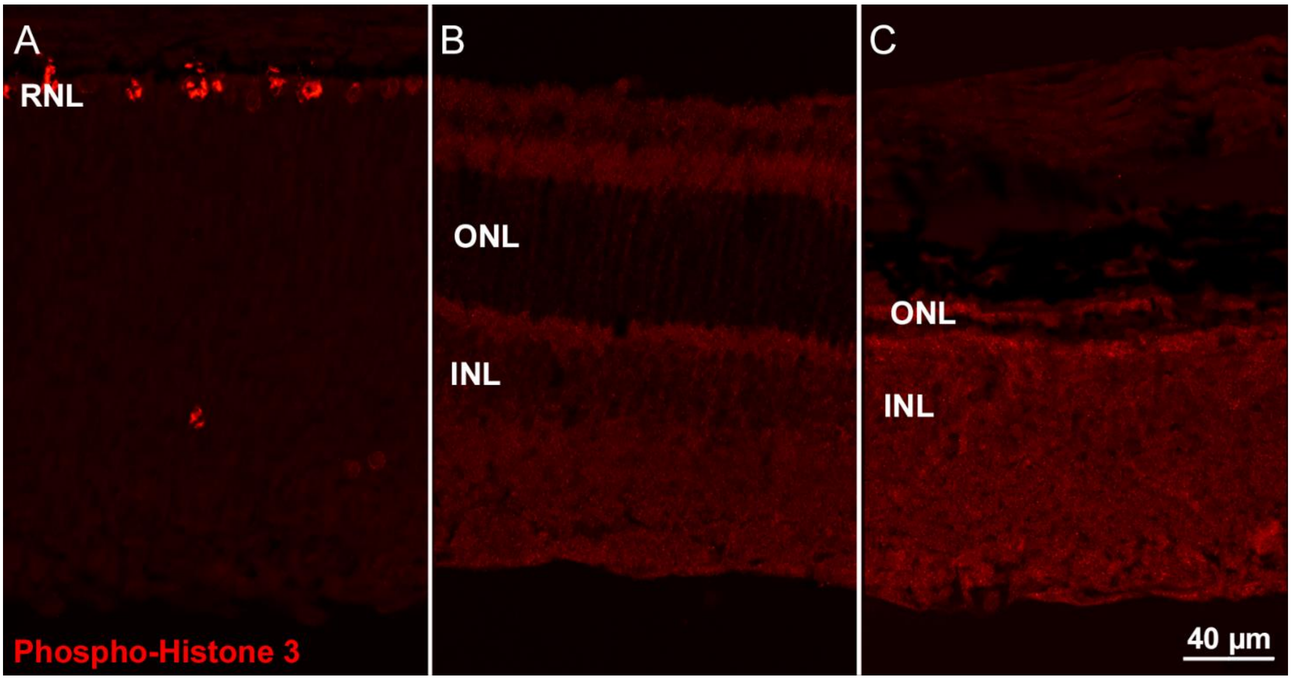

Supplementary Figure S5: CNF1 does not induces mitosis in retina. Phospho Histone 3 labeling of dividing cells detects, no active mitosis in vehicle or CNF1 treated retinas 7 days post injection. Retinas from a rat of 1 day of age (P1) was used as positive control. Dividing cells are visible in the retinoblastic layer (RNL).

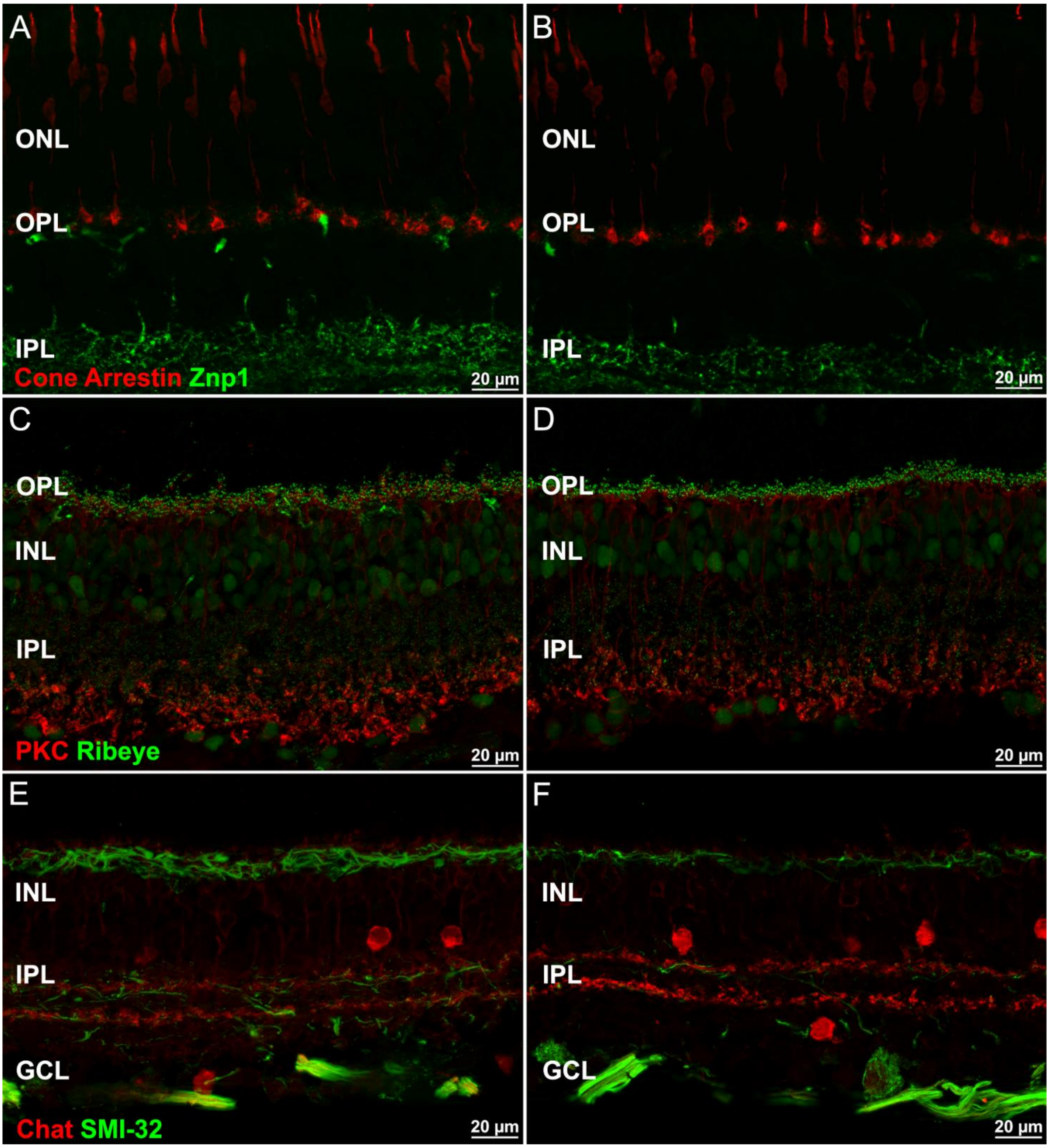

Supplementary Figure S6: Lack of morphological effects of CNFY 14 days after intraocular injection. Left side: Vehicle injected, control retinas (Panels A, C and E). Right side: CNFY treated samples (Panels B, D, and F). Treated and control retinas appear undistinguishable. A and B: cones are stained in red with cone arrestin antibodies; cone bipolar cells are stained in green with anti synaptotagmin 2 (Znp1) antibodies. C, D: rod bipolar cells are stained by PKC (red) and ribbon synapses by Ribeye (green). E and F Cholinergic amacrine cells are stained by Chat (red), while SMI-32 stains neurofilaments (green).
